# Methylarsonous Acid Transport by Aquaglyceroporins

**DOI:** 10.1289/ehp.8600

**Published:** 2005-12-02

**Authors:** Zijuan Liu, Miroslav Styblo, Barry P. Rosen

**Affiliations:** 1 Department of Biochemistry and Molecular Biology, Wayne State University, School of Medicine, Detroit, Michigan, USA; 2 Department of Nutrition, School of Public Health, and the Center for Environmental Medicine, Asthma, and Lung Biology, School of Medicine, University of North Carolina–Chapel Hill, Chapel Hill, North Carolina, USA

**Keywords:** arsenic trioxide, AQP9, aquaglyceroporin, methylarsonous acid

## Abstract

Many mammals methylate trivalent inorganic arsenic in liver to species that are released into the bloodstream and excreted in urine and feces. This study addresses how methylated arsenicals pass through cell membranes. We have previously shown that aquaglyceroporin channels, including *Escherichia coli* GlpF, *Saccharomyces cerevisiae* Fps1p, AQP7, and AQP9 from rat and human, conduct trivalent inorganic arsenic [As(III)] as arsenic trioxide, the protonated form of arsenite. One of the initial products of As(III) methylation is methylarsonous acid [MAs(III)], which is considerably more toxic than inorganic As(III). In this study, we investigated the ability of GlpF, Fps1p, and AQP9 to facilitate movement of MAs(III) and found that rat aquaglyceroporin conducted MAs(III) at a higher rate than the yeast homologue. In addition, rat AQP9 facilitates MAs(III) at a higher rate than As(III). These results demonstrate that aquaglyceroporins differ both in selectivity for and in transport rates of trivalent arsenicals. In this study, the requirement of AQP9 residues Phe-64 and Arg-219 for MAs(III) movement was examined. A hydrophobic residue at position 64 is not required for MAs(III) transport, whereas an arginine at residue 219 may be required. This is similar to that found for As(III), suggesting that As(III) and MAs(III) use the same translocation pathway in AQP9. Identification of MAs(III) as an AQP9 substrate is an important step in understanding physiologic responses to arsenic in mammals, including humans.

Trivalent inorganic arsenic [As(III)] is a known human carcinogen, with liver, skin and lung as target sites (Abernathy et al. 1999). In solution at physiologic pH, As(III) is primarily in the form of the undissociated acid arsenic trioxide [As(OH)_3_] and not the oxyanion arsenite (Ramirez-Solis et al. 2004). The anhydrous form of arsenite trioxide (As_2_O_3_) is also used clinically as a chemotherapeutic agent for the treatment of acute promyelocytic leukemia (Soignet et al. 1998). In tissues of some mammalian species, As(III) is methylated, forming trivalent and pentavalent products such as methylarsonous acid [MAs(III)], methylarsonic acid [MAs(V)], dimethylarsinous acid [DMAs(III)], dimethylarsonic acid [DMAs(V)], trimethylarsine oxide [TMAs(V)O] and trimethylarsine [TMAs(III)] (Cullen et al. 1984; Waters et al. 2004a). The liver is considered a major site of arsenic methylation, although other tissues also have the capacity to methylate arsenic (Vahter 2002). In humans, DMAs(III) and DMAs(V) appear to be the end products of this pathway (Thomas et al. 2001). MAs(III) and DMAs(III) are significantly more cytotoxic than As(III), perhaps because they are more potent inhibitors of critical enzymes (Thomas et al. 2001) or more efficient modulators of signal transduction pathways that regulate cellular metabolism and survival (Drobna et al. 2003; Walton et al. 2004). Thus, although the overall role of biomethylation in modulation of As(III) toxicity remains unclear, it is possible that MAs(III) and DMAs(III) significantly contribute to adverse effects associated with acute or chronic exposure to As(III).

A mammalian As(III) methyltransferase, Cyt19 or AS3MT, has been identified in rat liver (Lin et al. 2002; Walton et al. 2003). This enzyme catalyzes the sequential transfer of methyl groups from *S*-adenosylmethionine to trivalent arsenicals and the reduction of pentavalent methylated intermediates in the metabolic pathway for As(III) (Waters et al. 2004a, 2004b). The methylated arsenicals are released from liver into the bloodstream and end up in urine, skin, hair, and other tissues. For investigators to understand the mechanisms of arsenic toxicity and carcinogenesis as well as the ability of arsenic to serve as a chemotherapeutic agent, it is important to determine how methylated arsenicals are transported out of the liver and how they enter target tissues.

In this study, we show that aquaporin 9 (AQP9) conducts MAs(III). AQP9 is a member of the aquaglyceroporin family of channels that facilitates bidirectional movement of small neutral solutes such as glycerol and urea (Agre and Kozono 2003). Notably, the tissues with the highest expression of AQP9 include the liver (Abedin et al. 2002), an organ that plays a key role in the metabolism of arsenic. AQP9 is essential for the glycerol transport in the bile ducts and ductules of the liver. We previously identified the *Escherichia coli* aquaglyceroporin GlpF as a channel for As(III) and trivalent inorganic antimony [Sb(III)] (Meng et al. 2004; Sanders et al. 1997). In *E. coli*, uptake of metalloids produces toxicity. In contrast, in *Sinorhizobium meliloti*, downhill efflux of internally generated As(III) by another aquaglyceroporin, AqpS, confers resistance (Yang et al. 2005). Thus, aqualglyceroporins catalyze bidirectional movement of trivalent arsenicals. In *Saccharomyces cerevisiae*, the aquaglyceroporin Fps1p mediates uptake of As(OH)_3_, producing sensitivity. We have shown that rat AQP9, which has broadest solute permeability (Borgnia et al. 1999) among mammalian aquaglyceroporins, facilitates As(OH)_3_ transport when expressed in frog oocytes or *S. cerevisiae* (Liu et al. 2002). Thus, movement of arsenite appears to be a ubiquitous property of aquaglyceroporin channels from prokaryotes to eukaryotes.

In solution at neutral pH, the most likely form of MAs(III) is methanearsonous acid [CH_3_As(OH)_2_], so it was reasonable to consider the possibility that MAs(III) uses the same translocation pathway as As(OH)_3_. Here, we show that rat AQP9 conducts MAs(III). In contrast, yeast Fps1p did not exhibit significant MAs(III) conductivity, demonstrating that aquaglyceroporins have a wide variation in selectivity for arsenicals. We examined the roles of two specific residues in AQP9 in MAs(III) transport by site-directed mutagenesis. From the crystal structures of GlpF (Fu et al. 2000) and AQP1 (Sui et al. 2001), it has been proposed that the positive charge of GlpF residue Arg-195 and AQP1 residue Arg-206 serve as the filter that prevents movement of protons or other positive ions (Jensen et al. 2003; Tajkhorshid et al. 2002). In this study, we showed that substitution of AQP9 residue Arg-219 eliminates MAs(III) uptake, consistent with a requirement for an arginine residue for conduction of CH_3_As(OH)_2_. The hydrophobic residue Phe-56 in AQP1 has been proposed to orientate the water molecule to form a hydrogen bond with Arg-195 (de Groot and Grubmuller 2001). The effect of substitution of the corresponding residue in AQP9, Phe-64, was investigated, and the results indicate that a hydrophobic residue is not required at that position. We propose that AQP9 is central to arsenic metabolism in liver, and aquaglyceroporins may play similar roles in other tissues. As(III) enters the hepatocyte down its concentration gradient through AQP9. Inside the cell, As(III) is methylated and reduced to MAs(III). Internally generated MAs(III) then flows out of the hepatocyte and into the bloodstream down its concentration gradient via AQP9.

## Materials and Methods

### Strains and plasmids.

*E. coli* and *S. cerevisiae* strains and plasmids used in this study are described in Table 1. Two *E. coli* strains were used. The chromosomal *arsRBC* operon, which confers low-level resistance to arsenite, had been deleted to create the arsenic-hypersensitive strain AW3110 (Carlin et al. 1995). Strain OSBR1 was derived from AW3110 by inactivation of the *glpF* gene. Disruption of *glpF* makes the cells arsenite resistant again because arsenite cannot get into the cells (Sanders et al. 1997). Similarly, two *S. cerevisiae* strains were used. In yeast strain MG102, the *YCF1* and *ACR3* genes encoding arsenite resistance transporters were disrupted, rending the cells sensitive to arsenite (Ghosh et al. 1999). Strain HD9 was derived from MG102 by disruption of the *FPS1* gene (Liu et al. 2002), which makes the yeast cells resistant to arsenite because it is not taken up.

### Chemicals and media.

Methylarsine oxide provided by W.R. Cullen (University of British Columbia, Vancouver, Canada) was used as a precursor of MAs(III). Methylarsine oxide is hydrolyzed to MAs(III) in aqueous solution (Petrick 2001). Sodium arsenite was purchased from Sigma Chemical Co. (St. Louis, MO). *S. cerevisiae* strains were grown at 30°C in minimal synthetic dextrose (SD) medium (Adams et al. 1998) with 2% galactose and supplemented with the required auxotrophic requirements. *E. coli* cells were grown in Luria-Bertani (LB) medium (Sambrook et al. 1989) at 37°C.

### DNA manipulations and site-directed mutagenesis.

Plasmid pAQP9 carrying the rat *AQP9* gene (Liu et al. 2002) was purified using Qiagen miniprep spin column and used for site-directed mutagenesis (Stratagene, La Jolla, CA). Each mutation was verified by DNA sequencing of the entire gene. The mutants were transformed into yeast using a Geno easy-transform kit (Geno Technologies, St. Louis, MO). Oligonucleotides used were as follows: R219E forward, GGG ACT GAG GTC TTC AGC TGG GTT C; R219E reverse, GAA CCC AGC TGA AGA CCT CAG TCC C; R219H forward, GGG ACT GAG GTC ATG AGC TGG GTT C; R219H reverse, GAA CCC AGC TCA TGA CCT CAG TCC C. Construction of the R219A, R219K, F64A, F64W, and F64T mutants has been previously described (Liu et al. 2004). Expression of each mutant AQP9 protein was verified by sodium dodecyl sulfate polyacrylamide gel electrophoresis and immunoblotting, as described previously (Liu et al. 2004).

### Metal ion resistance assays.

For *E. coli*, strains were grown overnight at 37°C in liquid LB medium with shaking, diluted into LB with the indicated concentrations of metalloid, and allowed to grow with shaking for an additional 8 hr. For *S. cerevisiae*, strains were grown overnight at 30°C in liquid SD medium with 2% galactose and the appropriate supplements. The cultures were diluted into fresh SD media to an optical density at 600 nm (OD_600nm_) of 0.2 in the presence of varying concentrations of the metalloid salts and incubated for an additional 20 hr, after which growth was estimated from OD_600nm_. For growth on solid media, yeast strains were grown overnight at 30°C in liquid SD medium with 2% galactose. Equivalent numbers of cells from each culture were spotted in 10-fold dilutions with the indicated concentrations of As(III) or MAs(III). Growth was observed after 3 days of incubation at 30°C.

### Transport assays.

*In vivo* metalloid uptake assays were performed as previously described (Liu et al. 2002). Briefly, either *E. coli* or yeast cells were grown to exponential phase, harvested, washed with transport buffer consisting of 75 mM HEPES, 0.15 M KCl, 1 mM MgCl_2_, pH 7.3, and suspended to a density of 2 × 10^8^ cells/mL in the same buffer containing 0.1 M glucose, all at room temperature. The assay was initiated by addition of either 10 μM MAs(III) or 10 μM As(III), final concentration. Portions (0.1 mL) were withdrawn at intervals and filtered through nitrocellulose filters (0.5-μm pore size; Whatman Inc., Florham Park, NJ). The filters were washed with 5 mL of transport buffer 3 times and dried. The membrane was treated in 70% nitrate acid at 70°C until dissolved, and HPLC-quality water added to bring each sample to 7 mL. The amount of arsenic was determined by inductively coupled plasma mass spectrometry (ICP-MS) (PerkinElmer, Norwalk, CT).

## Results

### *MAs(III) uptake in* E. coli.

**Figure 1:**
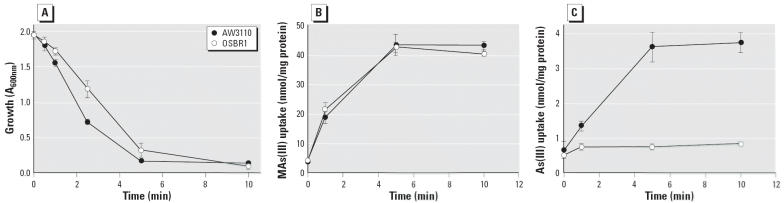
GlpF does not mediate MAs(III) uptake. (*A*) MAs(III) sensitivity in *E. coli* AW3110 (Δ*ars*) and OSBR1 (Δ*ars* Δ*glpF*). (*B*) Transport of MAs(III) by AW3110 and OSBR1. (*C*) Transport of As(III) by AW3110 and OSBR1. MAs(III) or As(III) was added at a final concentration of 10 μM. Each point represents the mean of three independent assays calculated using SigmaPlot 9.0 (Systat Software, Inc., Point Richmond, CA). Error bars indicate standard deviation.

We have previously shown that the aquaglyceroporin GlpF facilitates uptake of As(OH)_3_ in *E. coli* and that deletion of GlpF results in loss of uptake (Meng et al. 2004). Here we examined the ability of GlpF to transport MAs(III). As(III) and MAs(III) sensitivity and uptake were compared in two *E. coli* strains. One was AW3110 (Δ*arsRBC*), in which the chromosomal *arsRBC* operon, which confers resistance to both arsenate and arsenite, was deleted. Thus, this strain is hypersensitive to As(III). The second strain was OSBR1 (Δ*arsRBC*Δ*glpF*), which was derived from AW3110 by disruption of the gene for GlpF. Because this lacks the uptake system for As(III), it is resistant to the metalloid. Both strains were sensitive to MAs(III) (Figure 1A) and transported MAs(III) similarly (Figure 1B). These data suggest that most MAs(III) is taken up by a system or systems other than GlpF, which accounts for the sensitivity of *E. coli* to this methylated species. GlpF participation in MAs(III) transport, if any, is negligible. In contrast, AW3110 takes up As(III), whereas OSBR1 does not (Figure 1C). Because of the high rate of uptake of MAs(III), it is not possible to conclude whether GlpF conducts MAs(III).

### *MAs(III) uptake in* S. cerevisiae *by Fps1p and mammalian AQP9.*

**Figure 2:**
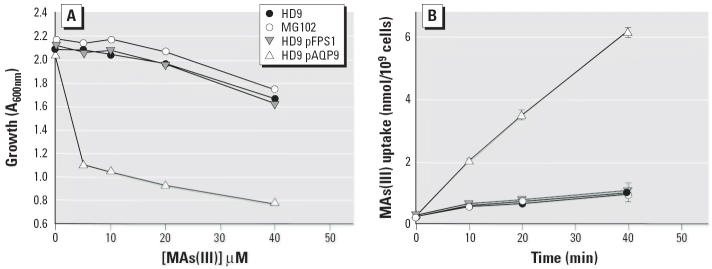
*AQP9* but not *FPS1* mediates MAs(III) uptake in yeast. (*A*) Sensitivity of strains MG102 (*acr3*Δ *ycf1*Δ), HD9 (*acr3*Δ *ycf1*Δ *fps1*Δ), and HD9 pFPS1 or pAQP9 to MAs(III). (*B*) Transport of MAs(III) by MG102 and by HD9 alone or with *FPS1* or *AQP9*. MAs(III) was added at final concentration of 10 μM. Each point represents the mean of three independent assays calculated using SigmaPlot 9.0. Error bars indicate standard deviation.

The ability of the yeast aquaglyceroporin Fps1p to conduct MAs(III) was examined. In these assays, *S. cerevisiae* strains MG102 (*acr3*Δ *ycf1*Δ) and HD9 (*acr3*Δ *ycf1*Δ *fps1*Δ) were used. MG102 is hypersensitive to As(III) because both *ACR3*, which encodes a plasma membrane As(III) extrusion carrier, and *YCF1*, which encodes a homologue of the ATP binding cassette ATPase multidrug resistance–associated protein (ABC ATPase MRP) (Cole et al. 1994) and sequesters arsenite triglutathione [As(GS)_3_] in the vacuole, were deleted (Ghosh et al. 1999). Like its parent, HD9 is *acr3*Δ *ycf1*Δ but has become resistant to As(III) because it is also *fps1*Δ and so does not take up As(III) (Liu et al. 2002). MG102 and HD9 exhibited similar resistance to MAs(III), as did HD9 expressing *FPS1* from a plasmid (Figure 2A). However, when rat AQP9 was expressed from a plasmid in HD9, yeast cells became sensitive to MAs(III).

**Figure 3:**
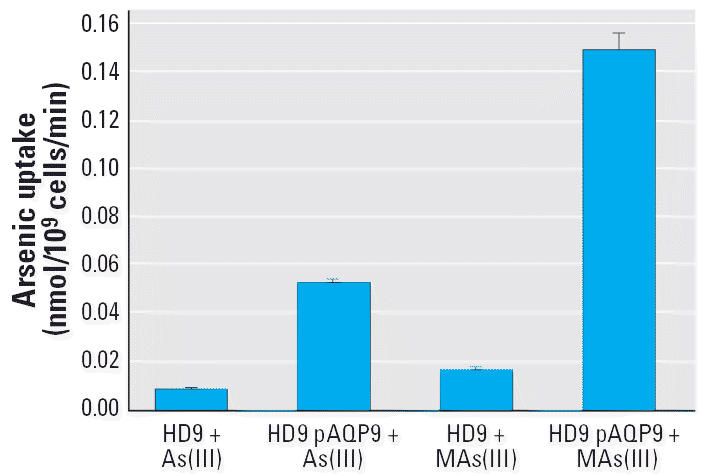
As(III) and MAs(III) uptake by AQP9 in yeast. Either As(III) or MAs(III) was added at a final concentration of 10 μM. Transport was assayed for 1 hr. Each bar represents the mean of three independent assays calculated using SigmaPlot 9.0. Error bars indicate standard deviation.

The resistance phenotype could be related to the ability of the cells to take up MAs(III). Cells of MG102, HD9, or HD9 pFPS1took up little MAs(III), but expression of rat AQP9 from a plasmid resulted in a high rate of uptake of MAs(III) in HD9 (Figure 2B). Because MG102 takes up considerably more As(III) than does HD9 (Liu et al. 2002), these results indicate that Fps1p facilitates uptake of As(III) but not MAs(III). In contrast, mammalian AQP9 takes up both inorganic and monomethylated trivalent arsenic. Strikingly, AQP9 conducts MAs(III) nearly 3 times faster than it does As(III) (Figure 3). These results indicate that AQP9 is more selective for the monomethylated form than for the inorganic form of trivalent arsenic, whereas Fps1p is highly specific for the inorganic form. The ability of AQP9 to conduct MAs(III) at a higher rate than As(III) is in good agreement with the results of recent studies that show considerably greater uptake and retention of MAs(III) than As(III) in cultured mammalian cells (Dopp et al. 2004; Drobna et al. 2005). Human AQP9 is 75% identical to and exhibits 86% overall similarity with the rat channel, and both transport As(III) with similar rates (Liu et al. 2004), so it is reasonable to expect that the human channel also conducts MAs(III). However, the human enzyme does not express well in yeast (Liu et al. 2004), so its ability to conduct MAs(III) was not tested here.

### Structure–function relationships in AQP9 transport of MAs(III).

**Figure 4:**
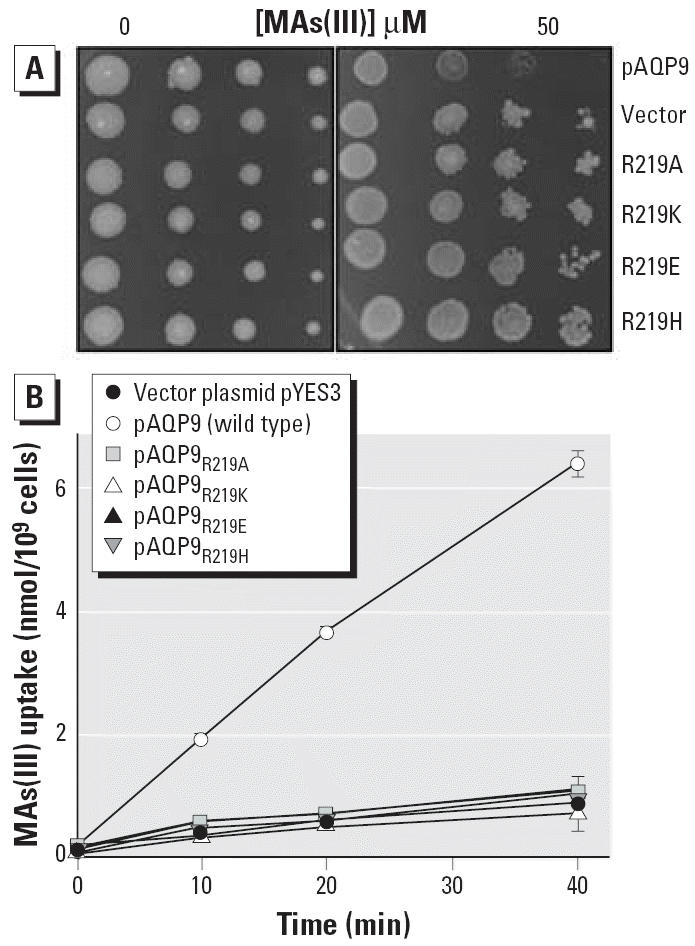
AQP9 Arg-219 is required for MAs(III) transport. (*A*) Complementation of MAs(III) sensitivity by Arg-219 mutants. MAs(III) was added in solid SD medium at 50 μM. (*B*) MAs(III) uptake in Arg-291 mutants. MAs(III) transport was assayed in strain HD9 expressing Arg-219 mutants at 10 μM MAs(III). Each point represents the mean of three independent assays calculated using SigmaPlot 9.0. Error bars indicate standard deviation.

We addressed the question of selectivity in aquaglyceroporins by mutagenesis of residues predicted to be involved in this process, including Arg-219, Trp-48, and Phe-200 of AQP9. To examine the requirement for a positive charge for MAs(III) uptake, Arg-219 was changed to alanine, lysine, glutamate, and histidine. Similar amounts of wild-type and mutant AQP9s were detected in the yeast membranes by immunoblotting (data not shown). HD9, which lacks the *FPS1* gene, is resistant to MAs(III) (Figure 4A) because it cannot take up the methylated metalloid (Figure 4B). Expression of wild-type mouse AQP9 renders cells of HD9 sensitive to MAs(III) (Figure 4A) because the cells take up MAs(III) (Figure 4B). Cells expressing any of the mutants remained resistant to MAs(III) (Figure 4A) and were unable to take up the organic arsenical (Figure 4B). Although loss of function in mutants could result from protein misfolding, these results are consistent with an arginine residue being required at position 219 for uptake of MAs(III) and suggest that MAs(III) shares the same channel pathway as As(III) and glycerol.

**Figure 5:**
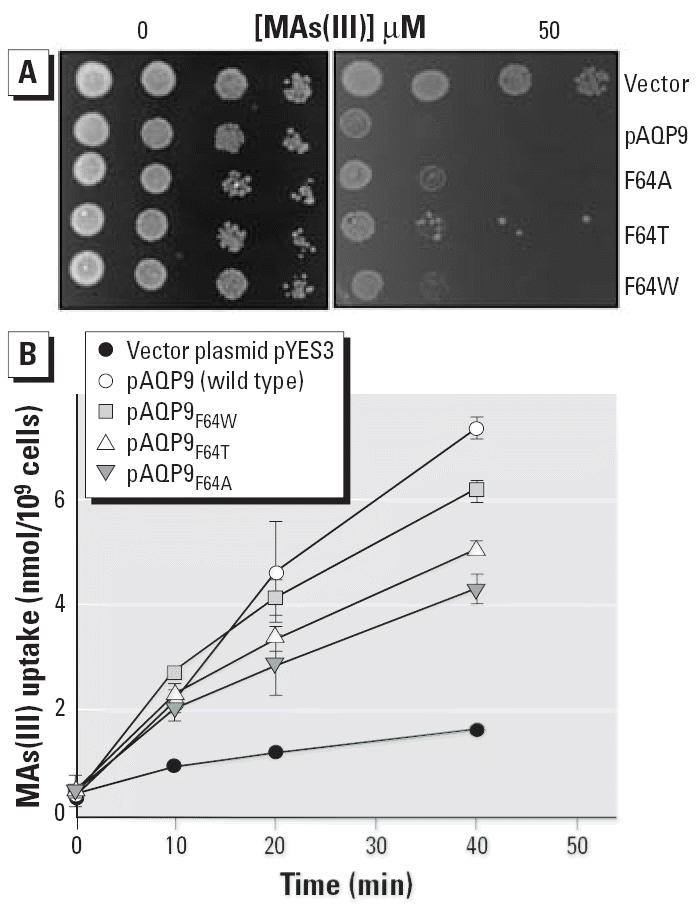
AQP9 Phe-64 need not be hydrophobic for MAs(III) uptake. (*A*) Complementation of MAs(III) sensitivity by Phe-64 mutants. MAs(III) was added in solid SD medium at 50 μM. (*B*) MAs(III) uptake in Phe-64 mutants. MAs(III) transport was assayed in strain HD9 expressing Phe-64 mutants at 10 μM MAs(III). Each point represents the mean of three independent assays calculated using SigmaPlot 9.0. Error bars indicate standard deviation.

We have previously shown that F64A, F64T, and F64W AQP9s are expressed in yeast and conduct As(III) and glycerol as well as wild-type AQP9 (Liu et al. 2004). Each mutant was transformed into yeast strain HD9, and both MAs(III) sensitivity and uptake was compared with HD9 transformed with wild-type AQP9. Again, with only vector plasmid pYES3, HD9 was resistant to MAs(III) and did not accumulate the metalloid, whereas wild-type AQP9 facilitated MAs(III) uptake and hence rendered the cells sensitive (Figure 5). Yeast cells expressing each of the Phe-64 mutants was as sensitive to MAs(III) as cells expressing wild-type AQP9 (Figure 5A). Cells expressing each mutant accumulated MAs(III), although at a somewhat lower rate than wild type (Figure 5B). Although a hydrophobic channel lining had been proposed to be required for AQP function, these results demonstrate that a hydrophobic residue at position 64 is not required for MAs(III) transport.

## Discussion

Aquaglyceroporins have been shown to facilitate uptake of As(III), including *E. coli* GlpF (Sanders et al. 1997), *S. cerevisiae* Fps1p (Liu et al. 2002; Wysocki et al. 2001), mouse AQP7 (Liu et al. 2002), and AQP9 from rat (Liu et al. 2002) and humans (Liu et al. 2004). Once inside human cells, As(III) is methylated to a variety of species, of which the monomethylated form represents a significant fraction of total arsenic found in most tissues (Hughes et al. 2003; Kenyon et al. 2005). The primary site of methylation is liver, but other organs such as kidney or testes may also methylate As(III) (Healy et al. 1998). The final fate of the methylated species is excretion, both in urine and in feces. How these compounds find their way from liver to other tissues such as blood, kidney, or cecum is not certain, and the routes of efflux of methylated arsenical from hepatocytes and uptake into other cell types are unknown. In solution at physiologic pH, inorganic trivalent arsenic is As(OH)_3_ (Ramirez-Solis et al. 2004). We would predict that, in solution, the monomethylated species would be CH_3_As(OH)_2_, molecularly similar to but less polar than As(OH)_3_. Because As(OH)_3_ is a substrate of aquaglyceroporins, we considered the possibility that those channels would also conduct CH_3_As(OH)_2_. The answer was unexpected in that the aquaglyceroporins exhibit selectivity in their ability to facilitate transport of trivalent arsenicals. AQP9 transports both As(OH)_3_ and CH_3_As(OH)_2_ but is more selective for the latter. In contrast, neither GlpF nor Fps1p exhibited any ability to facilitate CH_3_As(OH)_2_ transport and yet are able to transport As(III) as well as they do glycerol. It is interesting to note that *E. coli* possesses an endogenous uptake system for MAs(III), whereas yeast does not.

What governs the selectivity of the aquaglyceroporins for different trivalent arsenicals? In particular, what allows AQP9 to conduct MAs(III) when at least two other aquaglyceroporins cannot? The structures of GlpF and the strict water channel AQP1 have been determined at atomic resolution. The spacing at the narrowest region of the pore (the aromatic/arginine ring) is significantly wider in GlpF [3.4 Å (Nollert et al. 2001)] compared with AQP1 [2.8 Å in AQP1 (Sui et al. 2001)], which accounts for the broader selectivity of the aquaglyceroporin. As(OH)_3_ is a symmetrical molecular with an O—O distance of approximately 3 Å, just small enough to fit through the GlpF channel. CH_3_As(OH)_2_ is asymmetric compared with As(OH)_3_, and the As—C bond is longer than the As—O bond, which may account for the selectivity of GlpF. Although its channel diameter is not known, AQP9 transports a much wider range of substrates than does GlpF, suggesting that larger molecules may be able to traverse its channel.

Little is known about the relationship of individual residues to the function of AQP9. In members of the AQP superfamily, there is often a conserved arginine after the second asparagine–proline–alanine motif (Borgnia et al. 1999). From analysis of the crystal structures of AQP1 and GlpF, it has been proposed that the positive charge of this arginine residue forms an electrostatic triangle with two negatively charged residues to polarize the substrate molecules and to provide a filter that prevents charged molecules from entering the channel. We have previously shown that a positive charge is required on the corresponding residue in AQP9 (Arg-219) for both As(III) and glycerol uptake (Liu et al. 2004). Our results suggest that an arginine residue is preferred at position 219. In the structure of GlpF, the aromatic rings of Trp-48 and Phe-200 are perpendicular to each other and form a hydrophobic corner that has been proposed to serve as a selectivity filter (Lee et al. 2004). Corresponding to Trp-48 in AQP1 is the hydrophobic residue Phe-56, which has been proposed to orientate the water molecule to form a hydrogen bond with Arg-195. Because mutagenesis of the corresponding hydrophobic residue in AQP9, Phe-64, was not deleterious, a hydrophobic residue at this position is not required.

**Figure 6:**
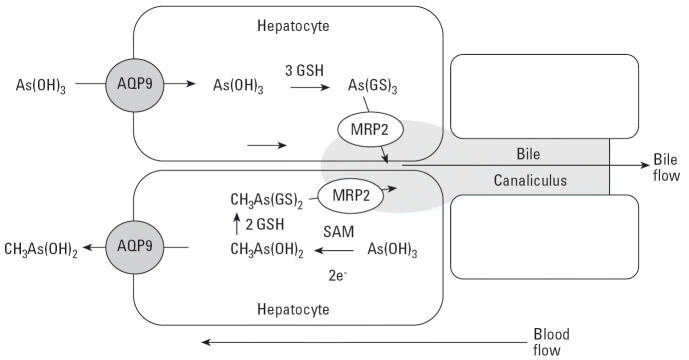
Proposed pathways of trivalent arsenic transport in liver. Abbreviations: GSH, glutathione; SAM, S-adenosylmethionine. Trivalent arsenic in the form of As(OH)_3_ flows down a concentration gradient from blood into hepatocytes through AQP9, which is the major aquaglyceroporin in liver (Carbrey et al. 2003). In the cytosol of the hepatocyte, As(III) can be either glutathionylated or methylated to MAs(V), which is reduced to MAs(III). As(GS)_3_ is pumped into bile by the MRP2 (Liu et al. 2001), and perhaps by other members of the ABC superfamily of ATPases. Alternatively, As(III) can be methylated and reduced to CH_3_As(OH)_2_, which then flows down its concentration gradient via AQP9 into blood.

AQP9 is highly expressed in liver (Abedin et al. 2002), where it plays an essential role in glycerol and urea transport (Carbrey et al. 2003). Because liver is also a key site for the metabolism of arsenic, we propose a model in which AQP9 catalyzes a key step in uptake of As(III) and efflux of MAs(III) (Figure 6). As(III) is taken up from the bloodstream by hepatocytes via AQP9. Inside the hepatocyte, it is methylated and reduced to MAs(III), which has a number of possible fates. It can be further methylated or glutathionylated. In mammals, both As(GS)_3_ and methylarsenite diglutathione [MAs(GS)_2_] are pumped into bile by multidrug resistance–associated protein 2 (MRP2) or homologues (Kala et al. 2000). Internally generated MAs(III) can also flow out of the cell down its concentration into the bloodstream. AQP9 expression in rat liver was induced up to 20-fold by fasting (Carbrey et al. 2003), suggesting that uptake of As(III) and redistribution of MAs(III) may be nutritionally responsive. Once in the bloodstream, MAs(III) can be redistributed into other tissues, including blood cells and kidney, where it is excreted. We predict that there are pathways for uptake and excretion of di- and trimethylated species (both tri- and pentavalent) as well, and it is important to identify their transport pathways. Identification and characterization of each transport system forms the basis for our future studies.

## Figures and Tables

**Table 1:** Strains and plasmids.

Strains/plasmids	Genotype/description	Source
*E. coli* strains		
AW3110	Δ*arsRB*(*C*):*cam* F^−^ IN(*rrnD-rrnE*)	Carlin et al. 1995
OSBR1	AW3110 *glpF*::Tn*phoA*, Km^r^	Sanders et al. 1997
*S. cerevisiae* strains		
MG102	*MAT*α *ura3-52 his6 leu2-3,112 his3-*Δ*200 trp1-901 lys2-801 suc2*Δ*ycf1::hisG acr3::URA3*	Ghosh et al. 1999
HD9	*MAT*α *ura3-52 his6 leu2-3,112 his3-*Δ*,200 trp1-901 lys2-801 suc2*Δ*ycf1::hisG acr3::URA3 fps1::leu2*	Liu et al. 2002
Plasmids		
pGEM-T	*E. coli* cloning vector, Ap^r^	Promega^a^
pYES3	*S. cerevisiae*–*E. coli* shuttle vector, Ap^r^, *TRP3*	Invitrogen^b^
pFPS1	1941-bp PCR fragment containing *FPS1* cloned in pYES3	Present results
pAQP9	1.1-kbp *Hin*dIII-*Kpn*I fragment containing AQP9 cloned into *Hin*dIII-*Kpn*I–digested pYES3	Liu et al. 2002

a Promega Corp., Madison, WI.

b Invitrogen Corp., Carlsbad, CA.
